# Disease outcomes with ublituximab in treatment-naïve participants: subpopulation analyses of the phase 3 ULTIMATE I and II studies in participants with relapsing multiple sclerosis

**DOI:** 10.3389/fimmu.2026.1771848

**Published:** 2026-05-29

**Authors:** Derrick Robertson, Enrique Alvarez, Lawrence Steinman, Hans-Peter Hartung, Peiqing Qian, Krzysztof Selmaj, Sibyl Wray, Daniel Wynn, Edward J. Fox, Koby Mok, Hari P. Miskin, Yihuan Xu, Christopher A. Garner, Bruce A. C. Cree

**Affiliations:** 1 University of South Florida, Tampa, FL, United States; 2 University of Colorado, Aurora, CO, United States; 3 Stanford University, Stanford, CA, United States; 4 Heinrich Heine University Düsseldorf, Düsseldorf, Germany; 5 Brain and Mind Centre, University of Sydney, Sydney, Australia; 6 Medical University of Vienna, Vienna, Austria; 7 Palacký University Olomouc, Olomouc, Czechia; 8 Swedish Medical Center, Seattle, WA, United States; 9 Center of Neurology, Lodz, Poland; 10 University of Warmia and Mazury, Olsztyn, Poland; 11 Hope Neurology, Knoxville, TN, United States; 12 Consultants in Neurology, Northbrook, IL, United States; 13 TG Therapeutics, Morrisville, NC, United States; 14 UCSF Weill Institute for Neurosciences, University of California San Francisco, San Francisco, CA, United States

**Keywords:** anti-CD20, disease-modifying therapy, monoclonal antibodies, multiple sclerosis, treatment naïve, ublituximab

## Abstract

**Background:**

Evidence suggests that treating relapsing multiple sclerosis (RMS) initially with a high-efficacy disease-modifying therapy (DMT) is superior to an escalating approach at reducing disability progression and relapse rate.

**Objectives:**

To evaluate the efficacy of ublituximab versus teriflunomide in participants without prior DMT (treatment-naïve population) and in a subset of treatment-naïve participants with symptom onset ≤ three years (early treatment subpopulation).

**Methods:**

Pooled *post hoc* analyses of ULTIMATE I/II were conducted at Week 96.

**Results:**

The annualized relapse rate was 0.081 for ublituximab (*n* = 345) versus 0.188 for teriflunomide (*n* = 377) (*p* < 0.001) in the treatment-naïve population and 0.130 for ublituximab (*n* = 139) versus 0.334 for teriflunomide (*n* = 140) in the early treatment subpopulation (*p* = 0.004). Twelve-week confirmed disability improvement rates were 10.7% versus 5.3% (*p* = 0.010) in the treatment-naïve population and 14.4% versus 3.6% (*p* = 0.002) in the early treatment population (ublituximab vs. teriflunomide). Significant reductions in gadolinium-enhancing T1 lesions and new/enlarging T2 lesions, respectively, occurred for ublituximab versus teriflunomide (treatment naïve: 0.031 vs. 0.791 and 0.390 vs. 4.144; *p* < 0.001 for both; early treatment: 0.051 vs. 1.030 and 0.508 vs. 6.068; *p* < 0.001 for both).

**Conclusions:**

Ublituximab was associated with significant treatment benefits at Week 96 in treatment-naïve participants and those receiving treatment within three years of symptom onset.

**Clinical trial registration:**

https://clinicaltrials.gov/study/NCT03277261 and https://clinicaltrials.gov/study/NCT03277248, identifiers NCT03277261 and NCT03277248.

## 1 Introduction

Older age and higher Expanded Disability Status Scale (EDSS) scores at baseline correlate with increased risk of progression in people with multiple sclerosis (MS) (1). Consistent with these findings, delay in treatment is associated with an increased likelihood of future disability progression and worsening symptoms in this population (1, 2). Early initiation of treatment with disease-modifying therapy (DMT) is recommended in people with active relapsing multiple sclerosis (RMS) to help minimize the early inflammation and neuronal damage that contribute to worsening progression and to potentially reduce the accumulation of disability (3–5). Evidence from observational studies suggests that initial treatment with a more efficacious DMT is superior to an escalating approach at reducing disability progression and relapse rate as well as increasing the likelihood of achieving no evidence of disease activity (NEDA) (6). The evidence of improved long-term outcomes following early use of high-efficacy therapy has supported a shift away from the escalation approach and toward early initiation of therapy with high-efficacy DMTs (6–9).

Ublituximab is a chimeric immunoglobulin G1 monoclonal antibody (mAb) that targets a unique epitope of CD20 and is glycoengineered for enhanced antibody-dependent cellular cytotoxicity (ADCC) (10). The exclusion of specific fucose molecules on the fragment crystallizable (Fc) region increases its affinity for all variants of Fc gamma receptor IIIa, thereby improving engagement of natural killer cells and resulting in increased ADCC relative to other anti-CD20 antibodies currently used to treat RMS (11). Ublituximab can be administered in lower doses and with shorter infusion times after the initial dose in people with RMS compared with other infused anti-CD20 mAbs (12, 13).

ULTIMATE I (NCT03277261) and ULTIMATE II (NCT03277248) were identical, Phase 3, randomized, multicenter, double-blind, active-control studies that evaluated the efficacy and safety of ublituximab versus teriflunomide in participants with RMS (10). These trials demonstrated a statistically significant reduction in annualized relapse rate (ARR) for ublituximab compared with teriflunomide over 96 weeks of treatment and significant improvements in the mean total number of gadolinium-enhancing (Gd+) T1 lesions and the number of new or enlarging T2 lesions. In a pooled analysis of data from the ULTIMATE I and II trials, rates of achieving NEDA-3 at Weeks 24–96 (re-baselined) were 3.6-fold higher among participants receiving ublituximab versus teriflunomide (14).

To explore the efficacy of early initiation of ublituximab therapy, *post hoc* analyses evaluated the efficacy of ublituximab in treatment-naïve participants enrolled in ULTIMATE I and II (defined as not receiving an approved DMT in the five years prior to study enrollment) as well as in the subset of treatment-naïve participants who received treatment within three years of symptom onset.

## 2 Methods

### 2.1 Study design and participants

*Post hoc* subpopulation analyses evaluated the efficacy and safety of ublituximab in treatment-naïve participants using pooled data from two identically designed clinical trials. The Phase 3 ULTIMATE I and II studies enrolled a total of 1094 adults with a diagnosis of RMS (relapsing-remitting or secondary progressive) with disease activity (10). Each study site’s institutional review board or ethics committee approved the trials, which adhered to Good Clinical Practice guidelines and the principles of the Declaration of Helsinki. Written informed consent was obtained from all participants. The studies included neurologically stable (≥30 days prior to screening and baseline) people aged 18–55 years with RMS (2010 revised McDonald criteria) (15) who had either at least two relapses in the past two years or one relapse and/or one or more Gd+ lesion in the previous year, brain magnetic resonance imaging (MRI) findings consistent with MS, and an EDSS score between 0–5.5 (inclusive) at screening (10). People were excluded from participation if they had a diagnosis of primary progressive MS, a disease duration ≥10 years with an EDSS score at screening of ≤2.0, or prior treatment with stem cell transplantation, alemtuzumab, leflunomide, natalizumab, teriflunomide, or an anti-CD20 antibody or other B-cell–targeted therapy. In the double-blind studies, participants were randomized to receive ublituximab 450 mg administered by one-hour intravenous infusion every 24 weeks (following Day 1 infusion of 150 mg and Day 15 infusion of 450 mg) and oral placebo or oral teriflunomide 14 mg once daily for 96 weeks and intravenous placebo.

### 2.2 Clinical and MRI endpoints

Clinical assessments (neurological examination, EDSS, and Multiple Sclerosis Functional Composite [MSFC]) were performed at screening, baseline, and every 12 weeks. The ARR was the number of confirmed, protocol-defined MS relapses per participant-year, where relapses were defined as new or worsening neurological symptoms due to MS only in the absence of fever or infection, lasting more than 24 hours, occurring after ≥30 days of neurological stability or improvement, and accompanied by objective neurological worsening consistent with an increase of at least 0.5 points in EDSS score, 2.0 points in one EDSS functional system score, or 1.0 point in each of ≥ two EDSS functional system scores. Each suspected relapse was centrally confirmed by an independent adjudication panel. Twelve-week confirmed disability improvement (CDI) was defined as a sustained and confirmed (≥12 weeks) reduction from baseline of ≥1.0 point in EDSS score (or ≥0.5 points when the baseline EDSS score was above 5.5) that was sustained and confirmed at least 12 weeks following initial documentation of neurological improvement. Twelve-week confirmed disability progression (CDP) referred to an increase from baseline EDSS score of ≥1.0 point (≥0.5 points when the baseline score was above 5.5) that was confirmed at least 12 weeks following initial documentation of neurological worsening and not caused by other factors such as fever, concurrent illness, or concomitant medication. Participants underwent brain MRI at Weeks 12, 24, 48, and 96. NEDA-3 status required having no confirmed relapses, no MRI activity (Gd+ T1 lesions/new or enlarging T2 lesions), and no 12-week CDP. Analyses of the primary and secondary efficacy endpoints for the treatment-naïve subgroup were prespecified in the master statistical analysis plan for the ULTIMATE studies.

### 2.3 Statistical analyses

Due to the *post hoc* nature of this analysis, tests are unadjusted, nominal, and descriptive, without adjustments for multiplicity.

Pooled *post hoc* analyses evaluated efficacy measures at Week 96 in participants who had not received an approved DMT in the five years prior to study enrollment (treatment-naïve population). Analyses on treatment-naïve participants who had their first MS symptom ≤ three years prior to enrollment (early treatment subpopulation) were also performed. Baseline characteristics and clinical endpoints (ARR, EDSS/CDI/CDP/NEDA, and MSFC) were analyzed in the modified intention-to-treat (mITT) population, defined as all participants who received at least one dose of trial drug and completed a baseline and at least one post-baseline efficacy assessment. The mITT population was prospectively defined in the study protocol and statistical analysis plan to minimize bias and was used for all previously reported analyses (10). Analyses of MRI endpoints were based on the mITT-MRI population, which included all participants administered at least one dose of trial drug who had a baseline and at least one post-baseline MRI assessment.

Baseline demographic and clinical characteristics were summarized with descriptive statistics. ARRs were expressed as least squares (LS) means and were based on a negative binomial model (generalized estimating equation [GEE]) for relapse count per participant with logarithmic link function, treatment, region, and baseline EDSS strata (≤3.5 or >3.5) as covariates, log (years of treatment) as offset (transforming the model from predicting the absolute count of relapses to predicting the annualized rate of relapses). Rate ratios for ublituximab versus teriflunomide were presented along with corresponding 95% confidence intervals (CIs). Mean changes from baseline in EDSS score by visit were compared between treatment groups using *t* tests. The number and percentage of participants with 12-week CDI and 12-week CDP at Week 96 were reported, with the hazard ratio (HR) (stratified) estimated using a Cox regression model with treatment group as the covariate and the *p*-value determined using a stratified log-rank test. Stratification factors were study, region, and baseline EDSS score.

Total numbers of Gd+ T1 and new or enlarging T2 lesions per MRI scan were analyzed with the use of negative binomial models (GEE) with logarithmic link function, treatment as covariate, and an offset based on the log-transformed number of post-baseline MRI scans. The LS mean change from baseline in MSFC score was analyzed via mixed model for repeated measures, with treatment, study, region, baseline EDSS strata, visit, treatment-by-visit interaction, and baseline value as covariates and using an unstructured covariance matrix, restricted maximum likelihood estimation, and the Satterthwaite method for degrees of freedom.

NEDA-3 rates (proportions of participants with NEDA-3, excluding those who discontinued treatment early for reasons other than death or lack of efficacy) at Weeks 24–96 (re-baselined) were presented along with odds ratios and corresponding 95% CIs, determined based on logistic regression models with treatment, study, region, baseline EDSS strata, and log-transformed baseline MRI lesion counts (T1 non-enhancing, Gd+, and T2) as covariates. EDSS progression events that occurred at the last scheduled visit (Week 96) were not considered to represent 12-week CDP because they could not be confirmed.

Missing data were not imputed for EDSS, MRI (Gd+ T1 and new or enlarging T2 lesions), or NEDA analyses. Missing data accounted for less than 0.5% of the total datasets for EDSS and MRI, and the missing data were balanced between the ublituximab and teriflunomide arms.

## 3 Results

### 3.1 Participant demographics and baseline characteristics

The ULTIMATE I and II randomized intention-to-treat population consisted of ublituximab (*n* = 546) and teriflunomide (*n* = 548). The overall mITT population consisted of 543 ublituximab-treated and 546 teriflunomide-treated participants. Baseline demographic and disease characteristics were comparable between ublituximab and teriflunomide treatment groups in both the treatment-naïve population and in the early treatment subpopulations (**Table 1**). Compared with the overall treatment-naïve population (defined as not receiving an approved DMT in the five years prior to study enrollment), participants in the early treatment subpopulation tended to be slightly younger, have slightly lower EDSS scores (mean difference of ≈0.5 points), and had more recently experienced symptom onset (mean difference of ≈ five years) and received an MS diagnosis (mean difference of ≈ three years).

**Table 1:** Participant demographics and baseline characteristics in treatment-naïve participants.

Characteristic	Treatment-naïve population		Early treatment subpopulation	
*Mean ± SD or %*	Teriflunomide (*n* = 377)	Ublituximab (*n* = 345)	Teriflunomide (*n* = 140)	Ublituximab (*n* = 139)
Age, years	35.7 ± 9.2	35.2 ± 8.6	32.5 ± 8.7	32.6 ± 8.5
Sex, female, %	63.7	61.4	64.3	64.0
Time since first MS symptoms, years	6.2 ± 5.7	6.4 ± 6.3	1.4 ± 0.8	1.5 ± 0.8
Time since diagnosis, years	3.9 ± 4.8	3.8 ± 5.0	0.7 ± 0.7	0.8 ± 0.7
Time since most recent relapse, months	6.0 ± 4.3	6.3 ± 3.8	5.0 ± 2.9	5.6 ± 3.2
Number of relapses in the year prior to screening	1.3 ± 0.7	1.3 ± 0.6	1.4 ± 0.7	1.5 ± 0.6
Number of relapses in the 2 years prior to screening	1.8 ± 0.9	1.8 ± 0.8	2.0 ± 0.9	1.8 ± 0.8
Baseline EDSS score	2.8 ± 1.2	2.8 ± 1.2	2.3 ± 1.0	2.2 ± 1.2
Number of baseline Gd+ T1 lesions	2.1 ± 4.7	2.4 ± 5.2	2.8 ± 5.5	2.4 ± 5.3
Number of baseline T2 lesions	62.5 ± 39.6	63.9 ± 39.5	52.7 ± 35.5	56.3 ± 40.0
T2 lesion volume, mL	14.4 ± 15.5	14.7 ± 14.7	9.4 ± 10.4	10.3 ± 10.5
Participants free of Gd+ T1 lesions at baseline, %	52.0	50.7	45.7	48.9

EDSS, Expanded Disability Status Scale; Gd+, gadolinium-enhancing; mITT, modified intention-to-treat; MS, multiple sclerosis; SD, standard deviation.

### 3.2 ARR

**Figure 1:**
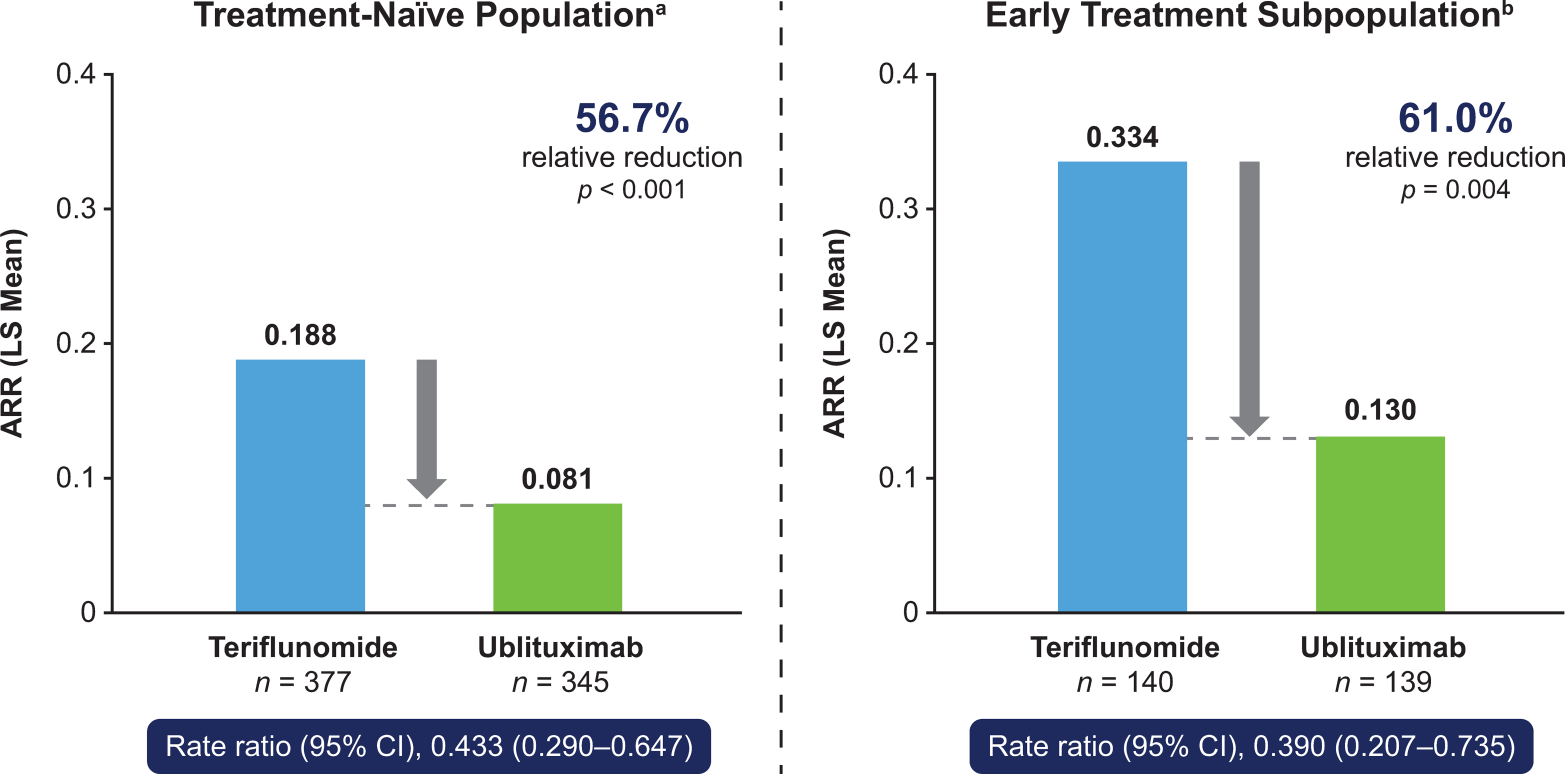
ARR at Week 96. ^a^Defined as participants who had not received approved DMT in the five years prior to study enrollment. ^b^Defined as treatment-naïve participants who had their first MS symptom ≤ three years prior to enrollment. mITT population. ARR, annualized relapse rate; CI, confidence interval; DMT, disease-modifying therapy; EDSS, Expanded Disability Status Scale; LS, least squares; mITT, modified intention-to-treat; MS, multiple sclerosis.

In the treatment-naïve population, ublituximab was associated with a 56.7% relative reduction in adjusted ARR compared with teriflunomide (0.081 vs. 0.188, respectively; *p* < 0.001; **Figure 1**). Similar results were seen in the early treatment subpopulation (61.0% decrease; 0.130 vs. 0.334, respectively; *p* = 0.004).

### 3.3 Disability improvement

**Figure 2:**
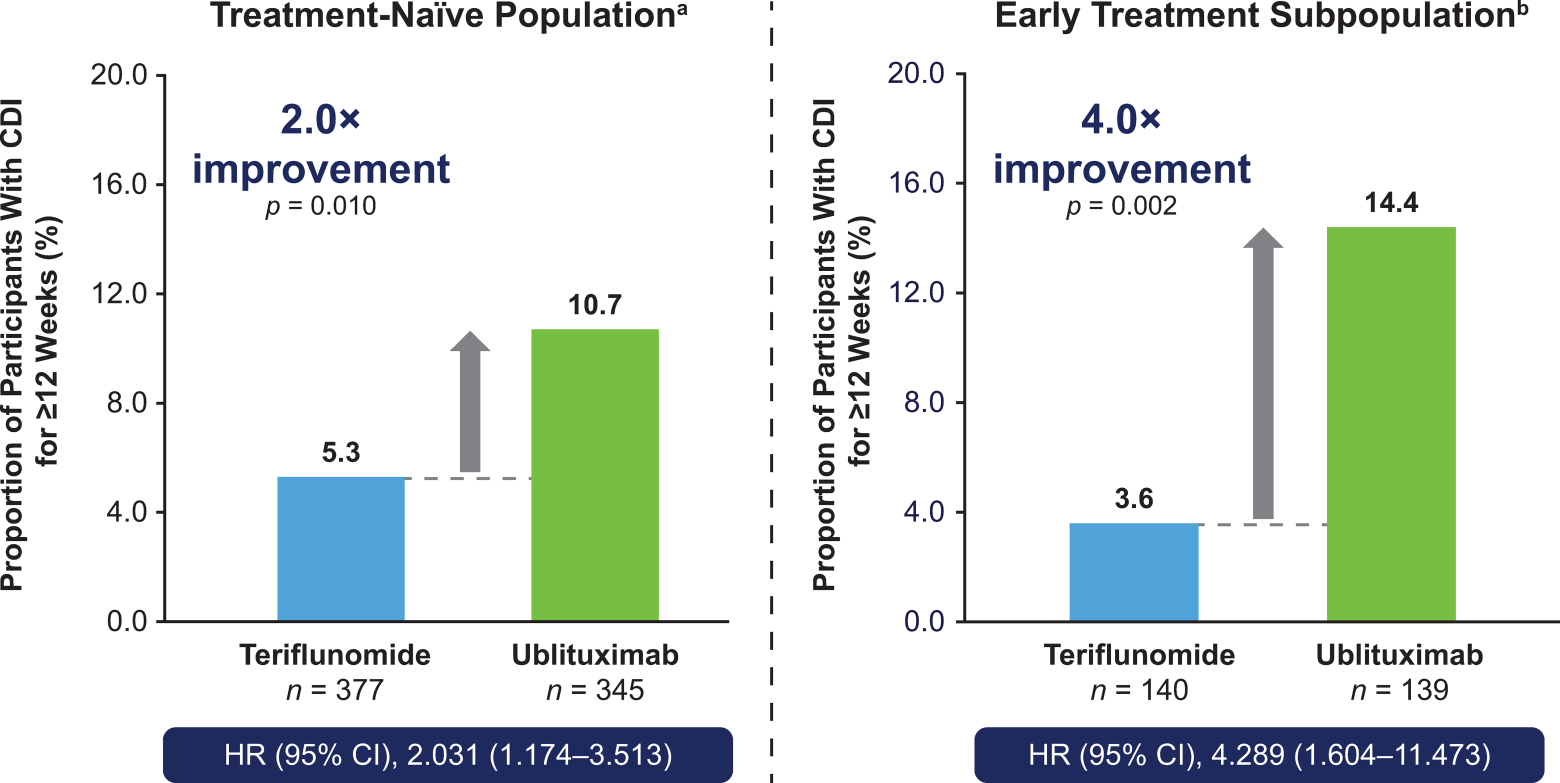
12-week CDI. ^a^Defined as participants who had not received approved DMT in the five years prior to study enrollment. ^b^Defined as treatment-naïve participants who had their first MS symptom ≤ three years prior to enrollment. mITT population. CDI, confirmed disability improvement; CI, confidence interval; DMT, disease-modifying therapy; HR, hazard ratio; mITT, modified intention-to-treat; MS, multiple sclerosis.

At Week 96, 2.0-fold more ublituximab-treated (10.7%) than teriflunomide-treated (5.3%) participants in the treatment-naïve population achieved 12-week CDI (HR 2.031, 95% CI: 1.174–3.513, *p* = 0.010) (**Figure 2**). In the early treatment subpopulation, a 4.0-fold greater proportion of participants in the ublituximab group had 12-week CDI compared with those receiving teriflunomide (14.4% vs. 3.6%, respectively; *p* = 0.002).

**Figure 3:**
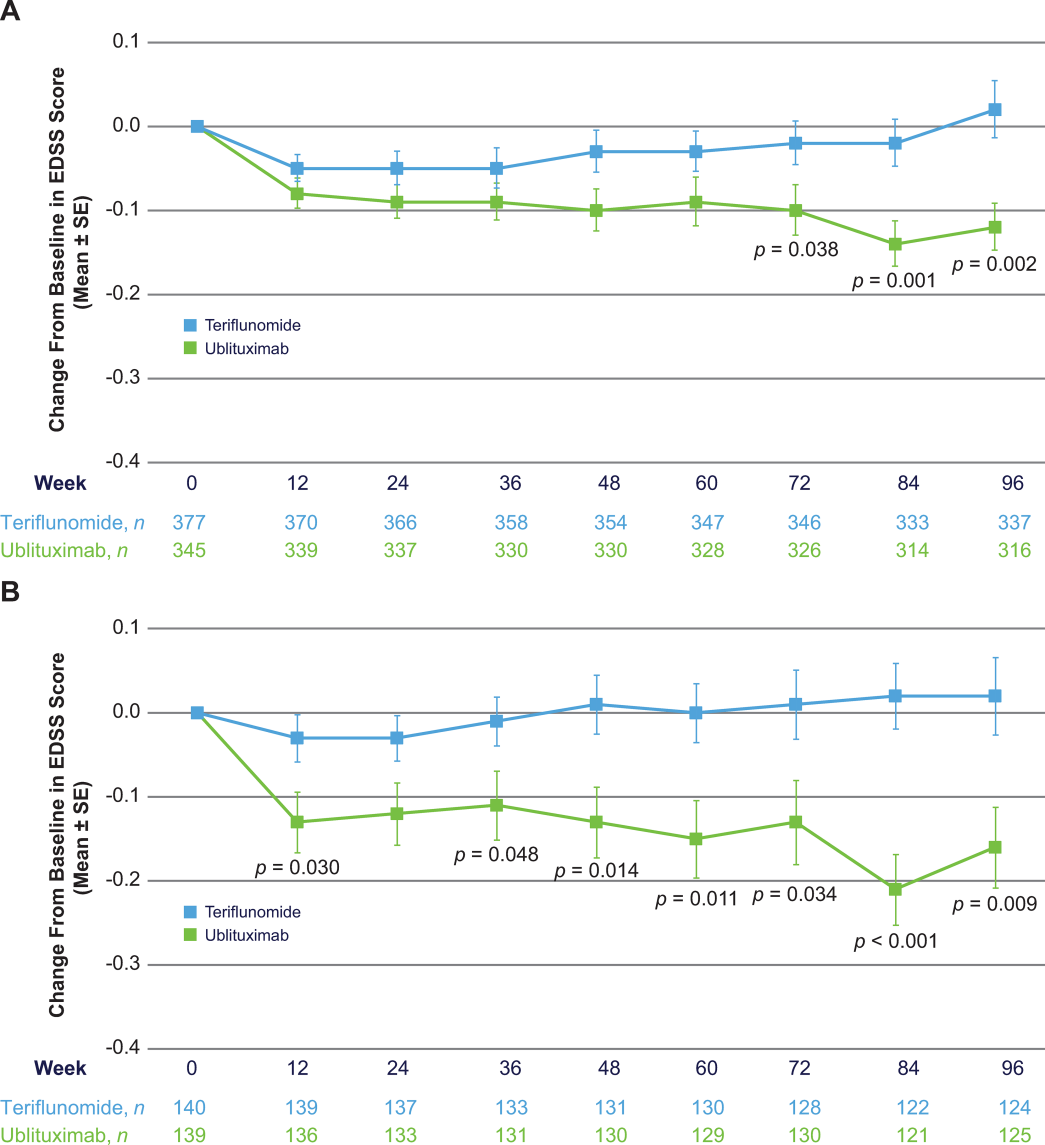
Mean EDSS score change from baseline in the **(A)** treatment-naïve population^a^ and **(B)** early treatment subpopulation^b^. ^a^Defined as participants who had not received approved DMT in the five years prior to study enrollment. ^b^Defined as treatment-naïve participants who had their first MS symptom ≤ three years prior to enrollment. mITT population. DMT, disease-modifying therapy; EDSS, Expanded Disability Status Scale; mITT, modified intention-to-treat; MS, multiple sclerosis.

Improvements in EDSS score from baseline were observed for ublituximab versus teriflunomide in both subpopulations at Week 96 and some earlier time points (**Figure 3**). As EDSS score change from baseline was not a prespecified endpoint of the ULTIMATE I and II studies, these data should be interpreted with caution.

### 3.4 Disability progression

The proportion of participants with 12-week CDP was low in both treatment groups in the treatment-naïve cohort (4.1% vs. 5.8%, ublituximab vs. teriflunomide; HR 0.698, 95% CI: 0.351–1.386, *p* = 0.297) as well as in the early treatment subpopulation (4.3% vs. 7.1%, ublituximab vs. teriflunomide; HR 0.514, 95% CI: 0.184–1.433, *p* = 0.196).

### 3.5 MRI activity

**Figure 4:**
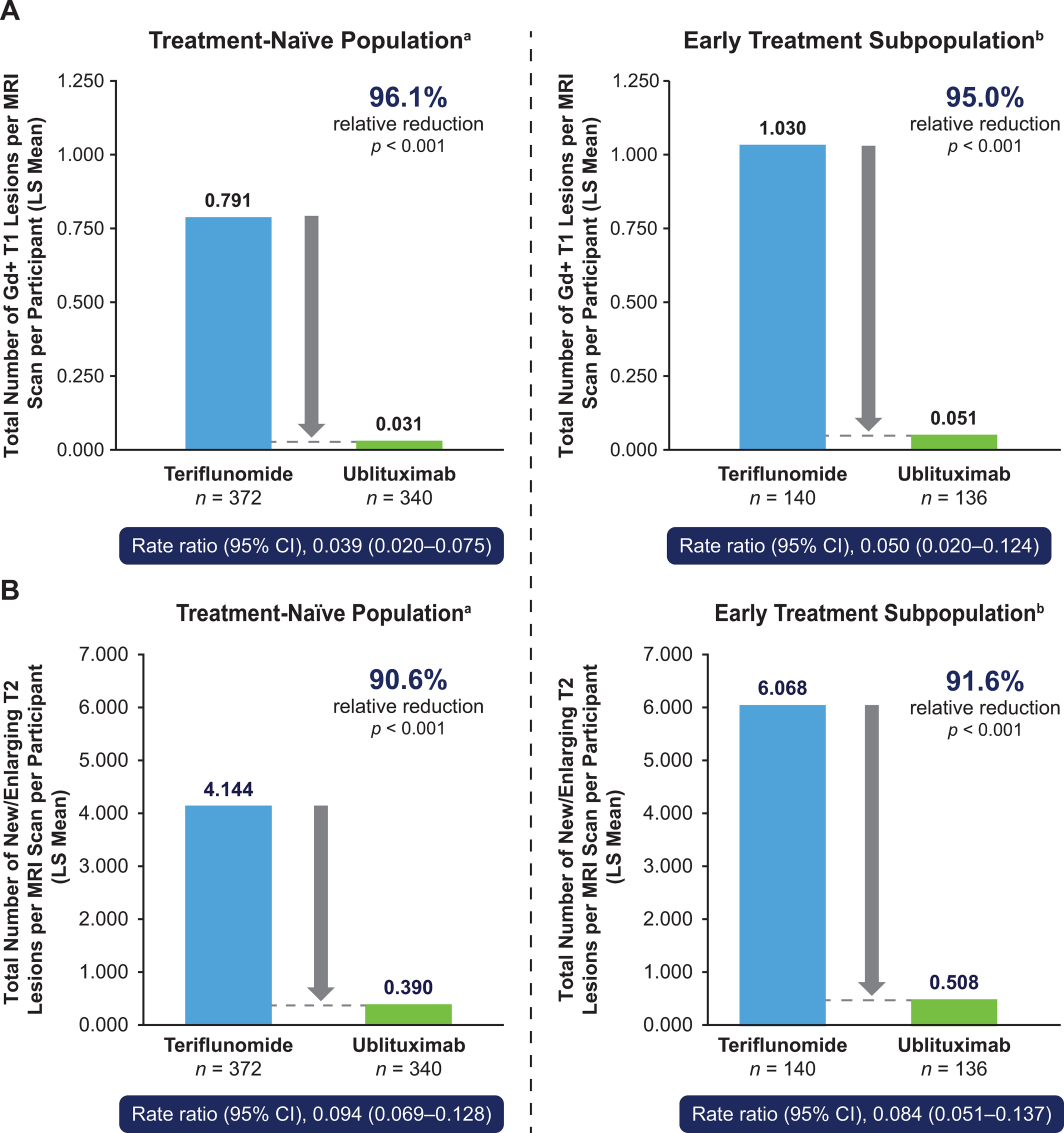
**(A)** Gd+ T1 lesions and **(B)** new/enlarging T2 lesions. ^a^Defined as participants who had not received approved DMT in the five years prior to study enrollment. ^b^Defined as treatment-naïve participants who had their first MS symptom ≤ three years prior to enrollment. mITT-MRI population. CI, confidence interval; DMT, disease-modifying therapy; Gd+, gadolinium-enhancing; LS, least squares; mITT, modified intention-to-treat; MRI, magnetic resonance imaging; MS, multiple sclerosis.

A statistically significant 96.1% relative reduction in Gd+ T1 lesions was observed with ublituximab versus teriflunomide in treatment-naïve participants (total number LS mean, 0.031 vs. 0.791; *p* < 0.001). (**Figure 4A**). Similar results were seen in the early treatment subpopulation (95.0% relative reduction; total number LS mean, 0.051 vs. 1.030; *p* < 0.001). Absolute reduction (95% CI) with ublituximab versus teriflunomide was -0.761 (-0.949 to -0.572) for the treatment-naïve group and -0.978 (-1.351 to -0.606) for the early treatment group.

The LS mean number of new or enlarging T2 lesions per scan was 90.6% lower with ublituximab compared with teriflunomide in the treatment-naïve population (total number LS mean, 0.390 vs. 4.144; *p* < 0.001) and 91.6% lower with ublituximab versus teriflunomide in the early treatment subpopulation (total number LS mean, 0.508 vs. 6.068; *p* < 0.001) (**Figure 4B**). Absolute reduction (95% CI) with ublituximab versus teriflunomide was -3.755 (-4.471 to -3.038) for the treatment-naïve group and -5.560 (-7.191 to -3.929) for the early treatment group.

### 3.6 Functional impairment

**Figure 5:**
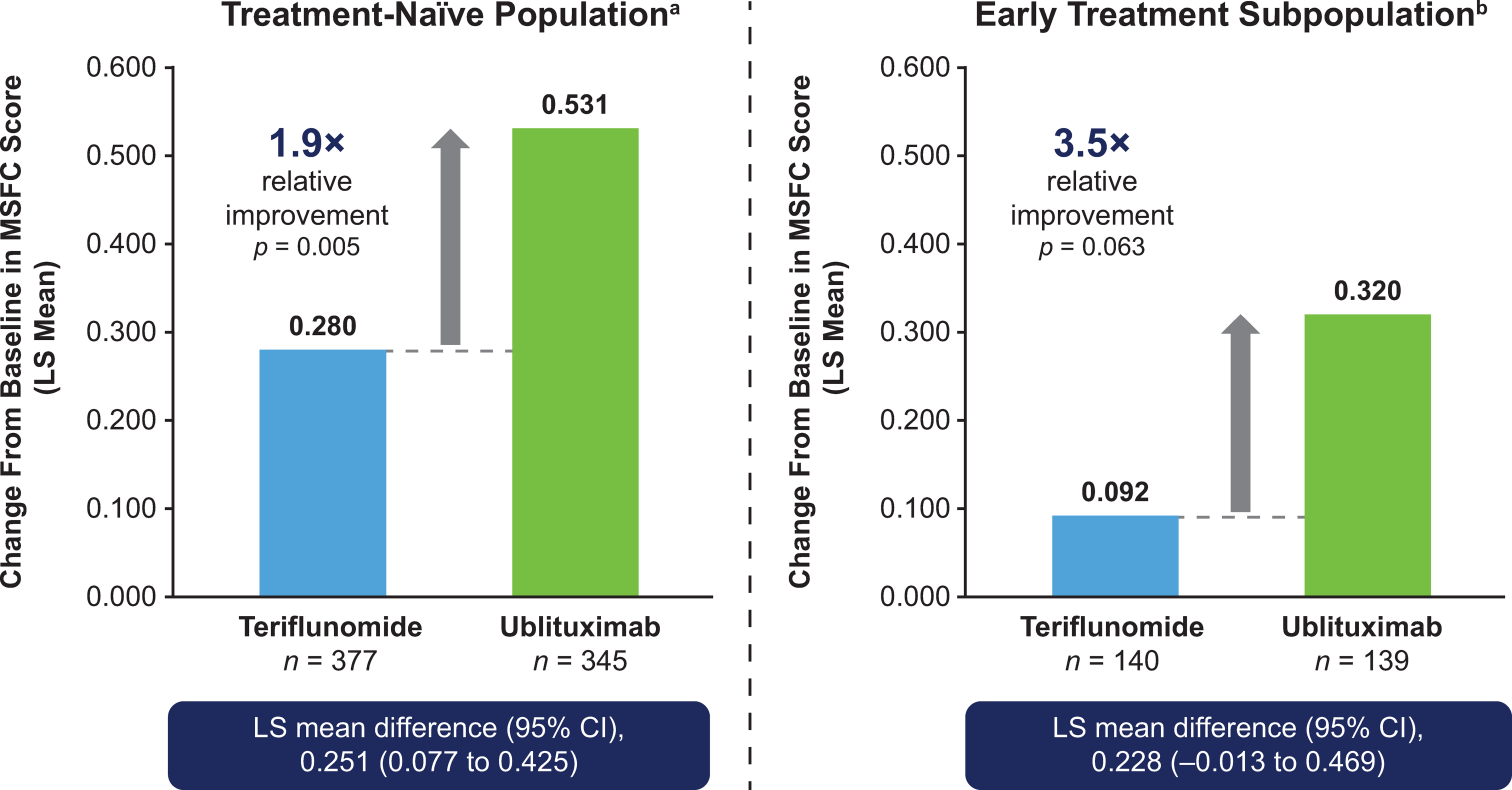
LS mean change from baseline in MSFC score. ^a^Defined as participants who had not received approved DMT in the five years prior to study enrollment. ^b^Defined as treatment-naïve participants who had their first MS symptom ≤ three years prior to enrollment. mITT population. CI, confidence interval; DMT, disease-modifying therapy; LS, least squares; mITT, modified intention-to-treat; MS, multiple sclerosis; MSFC, Multiple Sclerosis Functional Composite.

Among treatment-naïve participants, a 1.9-fold improvement in change from baseline in MSFC score was seen with ublituximab versus teriflunomide (LS mean, 0.531 vs. 0.280; *p* = 0.005) (**Figure 5**). The LS mean change from baseline in MSFC score in the early treatment subpopulation was 3.5-fold higher in participants receiving ublituximab versus teriflunomide (0.320 vs. 0.092), but the difference was not statistically significant.

### 3.7 NEDA

**Figure 6:**
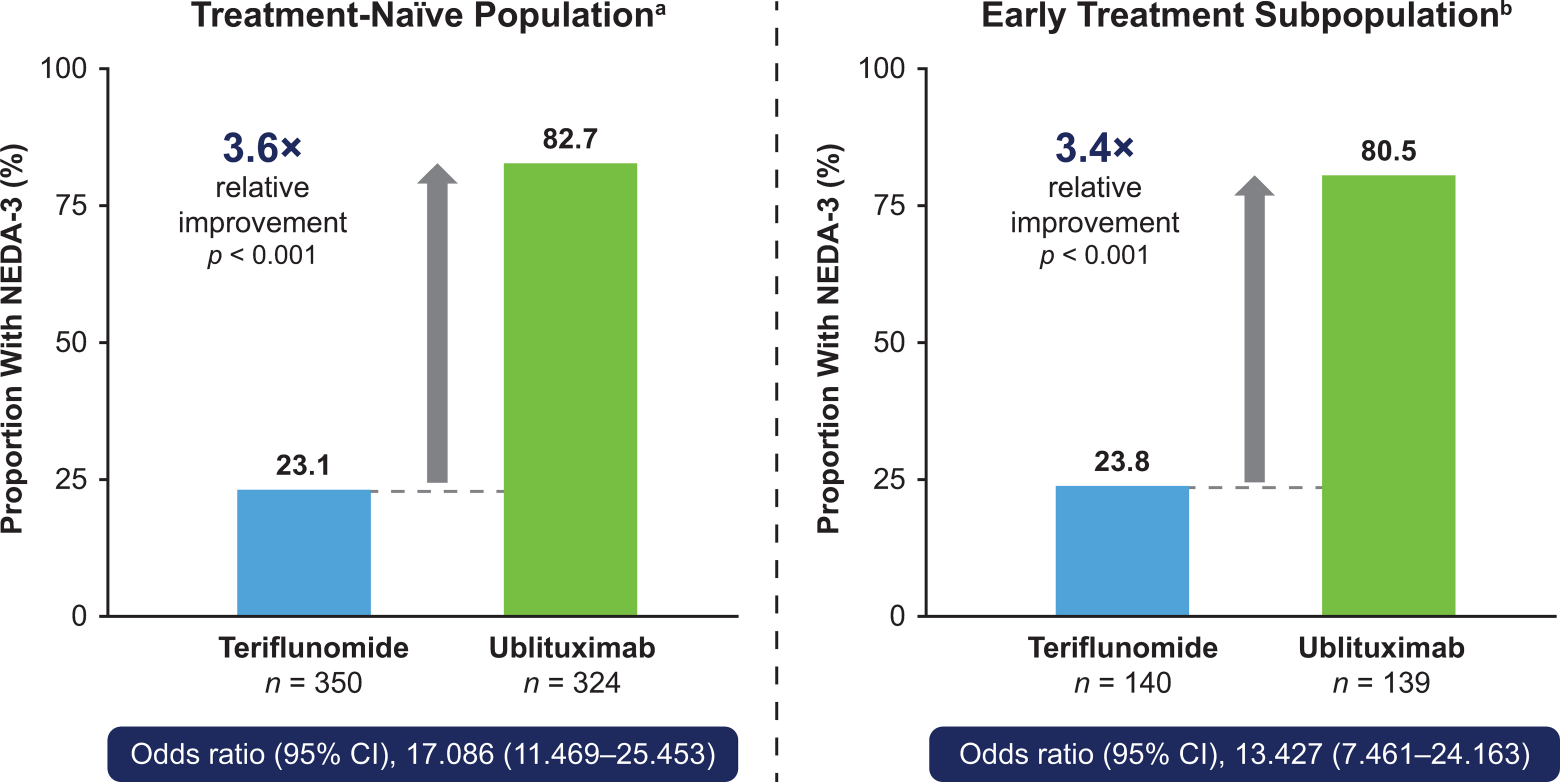
NEDA-3 at Weeks 24–96 (re-baselined). ^a^Defined as participants who had not received approved DMT in the five years prior to study enrollment. ^b^Defined as treatment-naïve participants who had their first MS symptom ≤ three years prior to enrollment. mITT population. Pooled *post hoc* analysis. NEDA-3 was defined as no confirmed relapses, no Gd+ T1 lesions, no new/enlarging T2 lesions, and no disability progression confirmed for at least 12 weeks. CI, confidence interval; DMT, disease-modifying therapy; Gd+, gadolinium-enhancing; mITT, modified intention-to-treat; MRI, magnetic resonance imaging; MS, multiple sclerosis; NEDA-3, three-parameter no evidence of disease activity.

NEDA rates at Weeks 24–96 (re-baselined) in the treatment-naïve subpopulation were significantly (3.6-fold) higher with ublituximab (82.7%) than with teriflunomide (23.1%) (*p* < 0.001) (**Figure 6**). Similar results were seen in treatment-naïve participants in the early treatment subpopulation: 80.5% with ublituximab versus 23.8% with teriflunomide (3.4-fold higher) (*p* < 0.001).

## 4 Discussion

Early disease progression in RMS is driven by focal inflammatory events, with faster disability progression occurring among individuals who experience more relapses or have more focal T2 lesions within the first few years after disease onset (16–18). Currently available DMTs are most effective early in the disease course during the period of peak central nervous system (CNS) inflammation (19). An extensive analysis of the Novartis-Oxford MS dataset, a large and comprehensive dataset in MS from ≈35,000 patients with up to 15 years of follow-up, demonstrated that treatment effects on disability progression were most effective in the youngest patients with lower disability levels and in patients at earlier disease stages (20). Emerging evidence suggests that early and initial treatment with high-efficacy DMTs may offer the best opportunity to target the early inflammatory process and protect against irreversible CNS damage and disability (6). In a meta-analysis of data from five observational studies with 1933 participants, initiation of therapy with a high-efficacy DMT was associated with a 30% reduction in EDSS score over five years when compared with an escalation strategy (*p* < 0.001) (21). Results of long-term extension studies show less disability progression and lower rates of NEDA among participants with continuous high-efficacy DMT exposure than among those initially randomized to a lower-efficacy DMT who then switched to the high-efficacy DMT, and these results also support the benefits of early intensification versus escalation therapy (22, 23). The evidence of improved long-term outcomes following early use of high-efficacy therapy has supported a shift away from the escalation approach and toward early initiation of high-efficacy DMTs (6–9). The expanding armamentarium of high-efficacy DMTs and the accumulation of evidence supporting the approach of early intensification has resulted in a shift over time in the US toward increasing use of high-efficacy DMTs in newly diagnosed people with RMS (24, 25).

Evidence from clinical trials is important to evaluate the efficacy of novel DMTs early in the course of disease. In these *post hoc* analyses of pooled data from ULTIMATE I and II, ublituximab was associated with significant treatment benefits across multiple efficacy measures at Week 96 versus teriflunomide in participants who had not received prior DMT. Compared with teriflunomide, ublituximab significantly reduced ARR by 56.8% (*p* < 0.001) after two years. In the overall ULTIMATE I and II study population, ARR continued to decrease over five years of ublituximab treatment; at five years, ARR was equivalent to one relapse every 50 patient-years (26). Relapses are associated with pain and reduced quality of life, psychological and mental status, and functional status (20, 27). Thus, relapse reductions can provide significant clinical benefits for patients in both the short and longer term.

A 2-fold greater proportion of participants with 12-week CDI (*p* = 0.010) and significantly greater improvement in EDSS score at Week 96 (*p* = 0.002) was observed for ublituximab versus teriflunomide in the treatment-naïve group. Ublituximab, compared with teriflunomide, also reduced the LS mean number of both Gd+ T1 and new or enlarging T2 lesions by ≥90% (both *p* < 0.001) and improved function based on an almost 2-fold difference in change from baseline MSFC score (*p* = 0.005). Rates of 12-week CDP were low (4%–6%) across treatment groups. At Weeks 24–96 (re-baselined), 82.7% of ublituximab-treated participants achieved NEDA-3 versus 23% of those receiving teriflunomide—a 3.6-fold difference (*p* < 0.001). Improvements with ublituximab versus teriflunomide on these outcomes were similar to, or greater than, those observed in the overall pooled ULTIMATE I and II population (10, 14, 26, 28).

Among treatment-naïve participants who had their first MS symptom within three years prior to enrollment in ULTIMATE I and II, ublituximab-related reductions relative to teriflunomide in relapse rate and MRI parameters were comparable with those in the overall treatment-naïve population. However, ublituximab produced greater improvements in disability relative to teriflunomide in the early treatment subpopulation than in the overall treatment-naïve population based on changes from baseline over time in EDSS scores and the proportion of participants with 12-week CDI. As with the overall treatment-naïve population, rates of 12-week CDP were low (4%–7%) over 96 weeks of treatment with ublituximab or teriflunomide. Notably, LS mean total numbers of Gd+ T1 lesions and new or enlarging T2 lesions at Week 96 were considerably lower in both the ublituximab and teriflunomide treatment groups in the early treatment subpopulation relative to the overall treatment-naïve population. The mean number of baseline Gd+ T1 lesions was similar for both groups of participants, although the mean number of baseline T2 lesions tended to be lower in the early treatment subpopulation and may partially explain the observation. NEDA rates were significantly improved with ublituximab versus teriflunomide in the early treatment cohort, similar to the overall treatment naïve population. These observations are consistent with greater efficacy of DMTs in the earlier stage of disease and highlight the importance of early diagnosis and initiation of treatment (19). However, in our study, the early treatment subpopulation represents a selected subset with shorter disease duration, and the observed differences could partly reflect different disease trajectories or measurement variability in smaller samples rather than differential treatment effects by disease duration. The objective of the analysis was not to demonstrate superiority of early versus late treatment, but to evaluate whether early treatment results in clinically meaningful results that could potentially translate to better long-term outcomes.

Despite potential limitations of these analyses (discussed below), the findings of improved efficacy outcomes with ublituximab versus teriflunomide in these subpopulations are consistent with the statistically significant improvements in ARR, Gd+ T1 lesions, and new or enlarging T2 lesions seen with ublituximab treatment in the full ULTIMATE I and II study population at 96 weeks (10).

The findings of the current analyses with ublituximab are consistent with the treatment benefits of other anti-CD20 mAbs in treatment-naïve people with MS overall and in early stage disease. Two identically designed Phase 3 trials (OPERA I and II) compared ocrelizumab with interferon (IFN)-β1a over 96 weeks in 1656 participants with RMS (29). In *post hoc* subgroup analyses of pooled data from OPERA I and II, ocrelizumab showed high levels of efficacy across measures of disease activity disability progression compared with IFN-β1a in treatment-naïve participants (30). In treatment naïve-participants initiating therapy two or more years after diagnosis, the benefits of an initial two years of ocrelizumab versus IFN-β1a treatment (followed by ocrelizumab treatment) were maintained at nine years of follow-up (31). A four-year, Phase 3, single-arm, open-label trial (ENSEMBLE) demonstrated sustained benefits of ocrelizumab in treatment-naïve participants with early stage (disease duration ≤ three years) RMS (32). Similarly, benefits of ofatumumab were shown in *post hoc* analyses of pooled data from ASCLEPIOS I and II (identically designed, Phase 3 trials comparing ofatumumab versus teriflunomide over 30 months in 1882 participants with RMS) (33) that evaluated the efficacy of ofatumumab relative to teriflunomide in 615 recently diagnosed (within 36 months), treatment-naïve participants (34). In this subgroup, ofatumumab significantly reduced ARR, Gd+ T1 lesions, new or enlarging T2 lesions, and six-month CDP compared with teriflunomide (34), with rate ratios/HRs similar to those observed in the overall ASCLEPIOS I and II population (33). As differences in study design and participant populations preclude comparisons between studies, prospective, randomized, head-to-head trials are warranted to compare the efficacy and safety of different high-efficacy DMTs early in the disease course in people with RMS.

Key limitations of the current analyses include the *post hoc* nature and the relatively small number of treatment-naïve participants with symptom onset within three years prior to enrollment. “Treatment naïve” was defined as having received no prior approved DMT in the five years before enrollment; therefore, we cannot exclude that some participants may have received earlier treatment. This could potentially affect baseline disease characteristics and potential disease trajectory with subsequent treatment. Some analyses in the early treatment population have low event rates (12-week CDI), further reducing statistical power. Additionally, regression to the mean has been noted in studies of people with MS, which could explain improvements seen in MRI activity; for example, in the present study. As such, these results must be considered as hypothesis generating rather than declarative, and all reports of statistical significance are nominal. While these analyses demonstrated sustained suppression of disease activity and progression over two years (≈96 weeks), longer-term data are important for understanding the benefits of therapy on the long-term disease course. The open-label extension of ULTIMATE I and II demonstrated sustained clinical benefits of ublituximab through five years of treatment (26). Notably, participants treated with continuous ublituximab exhibited a lower rate of disability progression and a higher probability of disability improvement at Year 5 compared with those initially treated with teriflunomide who switched to ublituximab after two years. This further supports the benefits of early initiation of high-efficacy DMTs, with the caveat that open-label extension data from participants who completed the controlled trial period can potentially introduce survivor bias and limit causal inference about treatment timing effects.

## 5 Conclusions

In pooled *post hoc* analyses of ULTIMATE I and II, ublituximab was associated with significant treatment benefits across multiple efficacy measures at Week 96 versus teriflunomide in participants who had not received prior DMT and among the subset of treatment-naïve participants with symptom onset within three years prior to study enrollment. These findings support the efficacy of ublituximab in these clinical populations.

## Data Availability

The datasets presented in this article are not readily available because access is subject to the protection of participant privacy, informed consent limitations, and applicable regulatory requirements. Requests to access the datasets should be directed to TGPublications@tgtxinc.com.
